# Study on the characteristics of future precipitation in response to external changes over arid and humid basins

**DOI:** 10.1038/s41598-017-15511-5

**Published:** 2017-11-09

**Authors:** Lianqing Xue, Boli Zhu, Changbing Yang, Guanghui Wei, Xianyong Meng, Aihua Long, Guang Yang

**Affiliations:** 1 College of Hydrology and Water Resources, Hohai University, Nanjing, 210098 P. R. China; 2 Shihezi University, Shihezi, 832003 P. R. China; 3 Jackson School of Geosciences, University of Texas at Austin, Austin, 78712 USA; 4 Hohai University Wentian College, Maanshan, 243000 P. R. China; 5 Xinjiang Tarim River Basin Management Bureau, Korla, 841000 China; 6 State Key Lab of Simulation and Regulation of Water Cycle in River Basin & China Institute of Water Resources and Hydropower Research, Beijing, 100038 China

## Abstract

The simulation abilities of the Coupled Model Inter-comparison Project Phase 5 (CMIP5) models to the arid basin (the Tarim River Basin, TRB) and humid basin (the Yangtze River Basin, YRB) were evaluated, determining the response of precipitation to external changes over typical basins. Our study shows that the future temporal and spatial variation characteristics of precipitation are different in different regions with the CMIP5. The annual and seasonal changes in precipitation were analyzed for the RCP2.6, RCP4.5 and RCP8.5 during 2021~2100 compared to those during 1961~2005. Precipitation shows an increasing trend in the TRB, but which decreases and then increases in the YRB, with a turning point in the middle of twenty-first Century. The ranges in annual precipitation increase with the increase in the scenario emissions in the future. Note that the Tarim River Basin is more vulnerable to the impact of emissions, especially for annual or spring and winter precipitation. Based on the uncertainty of CMIP5 data, the links between future precipitation changes and the elevation and relief amplitude were evaluated. The change of precipitation decreases with elevation, relief amplitude in the TRB, while it increases with elevation but decreases with relief amplitude in the YRB.

## Introduction

The global mean surface temperature have been undergone a long-term overall warming trend since the late 19th century^1,2^. Similar to increases in near-surface temperature, changes in precipitation may have significant impacts on ecology and environment^3^. Some of the changes observed in weather are projected to continue into the future^4^. The Intergovernmental Panel on Climate Change (IPCC), organized by World Climate Research Program (WCRP), provides climate data sets to the world leading climate-modeling groups for assessing climate change globally with numerical simulations^5^. Latest climate models in CMIP5 show that the changing climate will lead to changes in the intensity, extent and duration of extreme events, which may further trigger unprecedented impacts on the development of ecology, agriculture and environment^6–8^. With improvements in the representation of physical processes and the simulated fields^9^, the CMIP5 models performed better than the CMIP3 models^10,11^. Datasets of the CMIP5 models for the three Representative Concentration Pathways (RCPs) have been used in various studies, such as projections of precipitation, temperature, wind speed or sea surface temperature (SST)^6,11,12^.

While global climate models (GCMs) show obvious capabilities in projecting future climate, model parameters may have large uncertainties which depend on space and the forecast time horizon^13^. The uncertainties are due to the nature of the climate system itself with complex behaviors and large internal variability^1^. From a global perspective, the CMIP5 models well replicate the general feature^14,15^. At the regional scale, climate change effects are more complex, particularly at different environments and climate conditions. Such predictions are available from GCMs, but there exits large uncertainties from the model uncertainty, scenario uncertainty and internal variability^16^. Apart from the uncertainty caused by CMIP5 data, the topography of study area may result in the performances for precipitation by CMIP5 data^2,17–20^. Jiang, *et al*.^18^ concluded that most GCMs have topography-related cold biases and excessive precipitation. Su, *et al*.^2^ discussed the relation between the elevation of the study area and precipitation, temperature simulated by multi-model ensemble, who pointed out that the ensemble of CMIP5 underestimates the actual temperature and overestimates the actual precipitation in higher altitude regions over Indus River Basin. In this study, we focus on the influence of the topography of study area while simulating precipitation, particularly in elevation and relief amplitude.

This study focuses on two areas in China: the Tarim River Basin (TRB) with an arid climate and the Yangtze River Basin (YRB) with a humid climate, the Dry-Wet climate zones of which divided by aridity index combined with measured annual precipitation and annual potential evapotranspiration. The TRB is located in northwestern China, characterized by limited rainfall and high evaporation^21,22^, which is one of the world’s foremost endorheic drainage systems dominated by an arid inland climate^23^. The basin is typically supplied by precipitation and melting snow water from the cold mountainous area^24^. Precipitation at the mountainous area is recorded over 300 mm per year, while precipitation at the plain regions differs from 60 to 200 mm per year^25^. The studies about regional climate change over recent decades at the TRB have concluded an increasing trend in both the temperature and precipitation^26,27^. Yang, *et al*.^28^ reported drought months are projected to decrease by about 14% in the next decades, while the drought duration may be shorted to 3 months on average. The YRB is located in a humid area with abundant rainfall in China, where the spatial distribution of annual precipitation ranges from 270~500 mm in the northwest and 1600~1900 mm in the southeast^29^. Precipitation in summer accounts for 70~80% of its annual total amount for the East Asia monsoon^30^. Sang, *et al*.^31^ found an increasingly climate extremes and the accompanying server losses on economy over 1961~2010. During recent years, a wetting tendency is observed in the eastern Tibet Plateau and the middle and lower YRB, while the other regions experience precipitation deficits^30^. Many studies confirmed that the observed increase in precipitation at the YRB is mostly associated with an increase of high-intensity precipitation events which further lead to more frequent floods^17^. A significant positive trend in flood stream flow over the last 40 years were reported^32^. Flood events occur at the flood-prone areas almost every year in the last century^33^.

A number of studies have focused primarily on evaluating ability of the CMIP5 models to simulate precipitation and applicability to projection of potential precipitation changes at a particular basin or region. With the high-resolution daily GPD datasets over the period of 1961~2013,Wu, *et al*.^34^ showed a wetter trend at the TRB but drier conditions at the YRB. Although many studies about the climate change at each of the two basins have been reported in the literature, very few conducted a comparative analysis of precipitation historically and in the future at the two basin. Pan, *et al*.^33^ conducted a comparative analysis of historical and projected spatial-temporal distribution of extreme precipitations between the Mississippi basin in USA and YRB in China using 31 CMIP5 models. In this study, we chose the TRB and YRB, where are in almost opposed external environment conditions.

In order to facilitate the adaptive management to the climate change at different areas, it’s critical to investigate the implications of climate changes and estimate its temporal-spatial variation patterns^6,35,36^. Therefore the characteristics and response degree of future precipitation in typical watersheds to climate change should be analyzed. The main objectives of this study are to 1) evaluate 20 high-resolution CMIP5 models in terms of precipitation simulation for the Tarim River Basin and Yangtze River Basin, 2) project changes in both annual and monthly precipitation for the years 2021~2060 and 2061~2100 based on 1961~2005 for three RCPs: RCP2.6, RCP4.5 and RCP8.5, and 3) compare the differences and links between future precipitation changes and topographic features at the two basins.

## Results

### Model evaluation and downscaling

#### Model evaluation

The temporal processes and spatial distributions of precipitation in the TRB and YRB were analyzed from temporal and spatial scales respectively.

**Figure 1:**
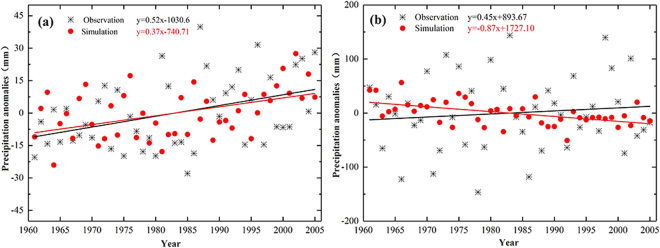
The temporal trend of precipitation anomalies of the observation and multi-model ensemble from 1961 to 2005 over the Tarim, (**a**) and Yangtze. (**b**) River Basin. (Note: black marks represent the annual observed precipitation, red dots represent the annual simulated precipitation, black lines represent annual observed precipitation tendency of the sequence, red lines represent annual simulated precipitation tendency of the sequence).

On temporal scale, the annual precipitation measured over the TRB in the baseline period is only about 1/10 of the YRB. The observed (simulated) annual precipitation is 100.45 mm (354.86 mm) over TRB, where exists a great deviation. The observed (simulated) annual precipitation is 1092.73 mm (1311.89 mm) over YRB, and the simulated bias is about 19.61% of the measured value. Figure 1 displays the temporal trend of annual precipitation anomalies of the observation and multi-model ensemble from 1961 to 2005 over the TRB (a) and YRB (b). The anomaly is defined as the original data subtracting the mean. The annual precipitation is increasing in both the observed data (5.2 mm per decade) and the CMIP5 ensembles (3.7 mm per decade) over TRB, which both show significant increasing trend. The annual precipitation is increasing in the observed data (4.5 mm per decade) over the YRB, while decreasing in the CMIP5 ensembles (−8.7 mm per decade). The small whole fluctuation range of simulated data shows that the simulated rainfall peak is weakened. GCMs data failed to represent the uncertainty factors of annual precipitation, which could have a great impact on the subsequent analysis of extreme precipitation events.

**Figure 2:**
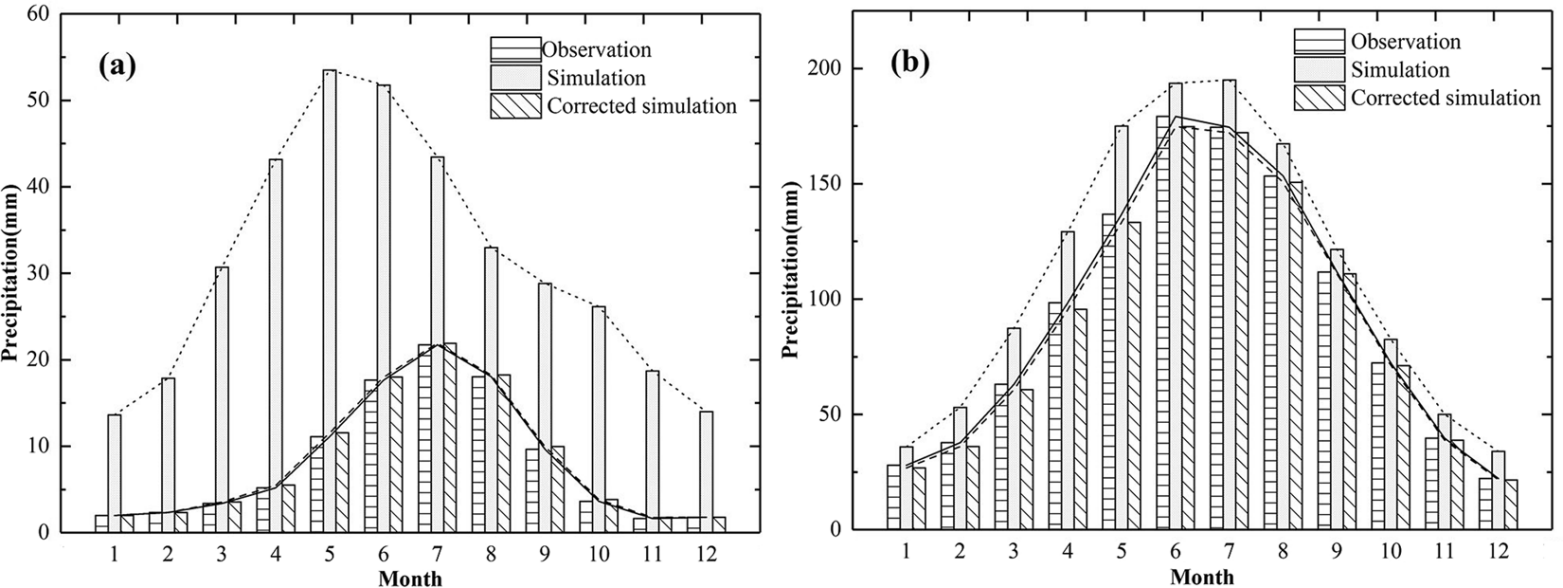
Monthly variation of observed, simulated and corrected precipitation during 1961~2005 over the Tarim, (**a**) and Yangtze. (**b**) River Basin. (Note: Black dotted lines are the linear fitting for the annual simulated precipitation during the 1961~2005, black lines are the linear fitting for the annual observed precipitation during the 1961~2005, black short dash lines are the linear fitting for the annual simulated precipitation during the 1961~2005).

From the monthly variation of observed and simulated precipitation (Fig. 2), the observed and simulated data both have sharp distinctions among seasons over two basins. Over TRB, the CMIP5 ensemble model, however, greatly overestimates the precipitation during each month and the observed (simulated) precipitation concentration period is June~August (May~July). The great bias between precipitation from CMIP5 and observed data has also been found in several studies. Fang, *et al*.^37^ found that the precipitation from CMIP5 in Xinjiang, China (including the Tarim River Basin) is much higher than the measured data from station by two times from 1962 to 2011. Over YRB, the CMIP5 ensemble model slightly overestimates the precipitation during each month and seasonal variation of precipitation is well captured.

**Figure 3:**
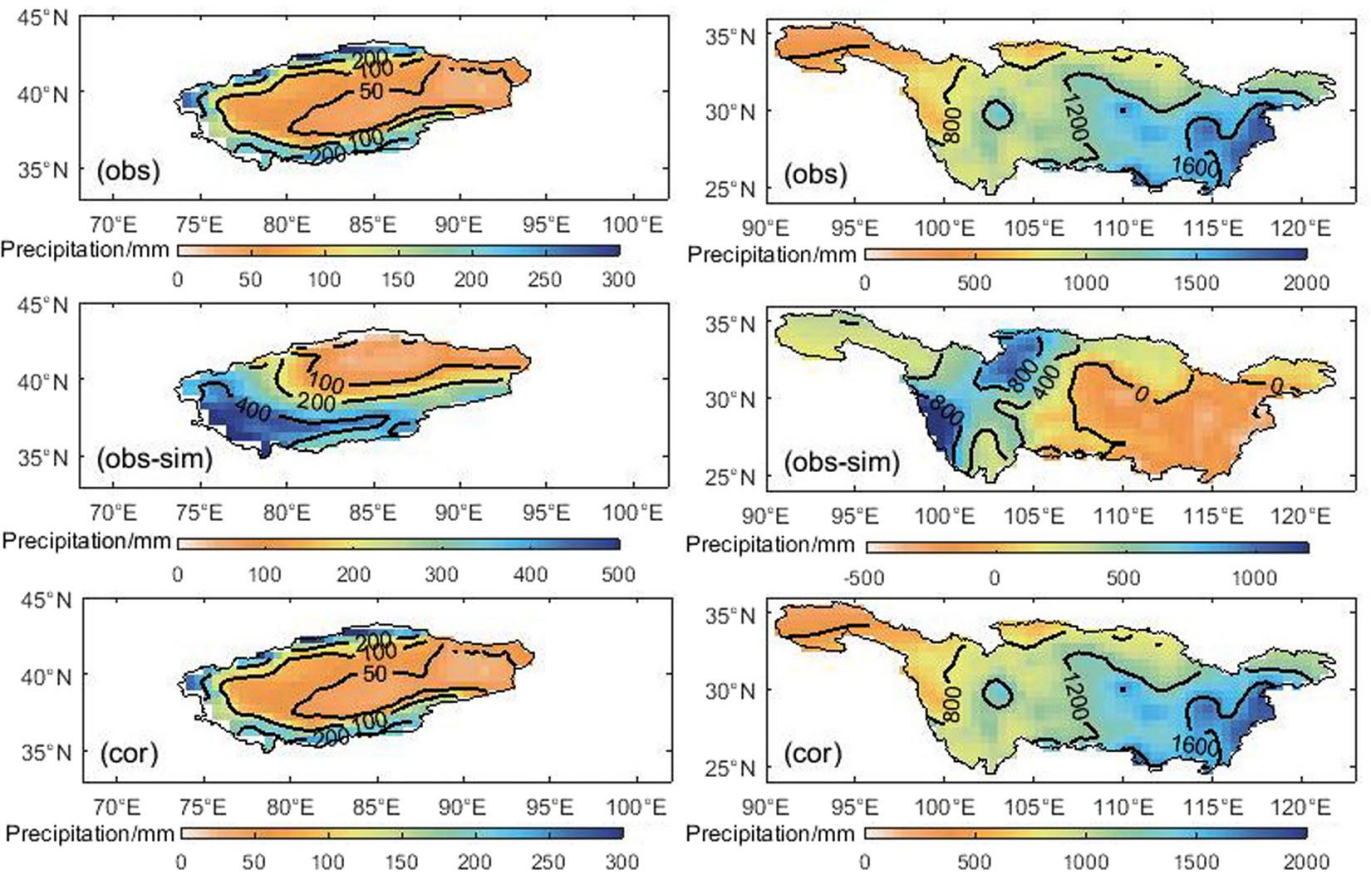
Spatial distribution of observation (obs), bias between observation and simulated precipitation (obs-sim), corrected simulated precipitation (cor) during 1961~2005 over the Tarim (the left panel) and Yangtze (the right panel) River Basin. (Note: the maps were generated with data available from the Chinese Geospatial Data Cloud using Matlab (version R2016a; https://cn.mathworks.com/).

On spatial scale, Fig. 3 displays the spatial distribution of observation (obs) and bias (obs-sim) between observation and simulated precipitation during 1961~2005 over the TRB and YRB. Over TRB, the observed precipitation is decreasing from outside to inside, which is well captured by the CMIP5 ensemble model. And the spatial correlation coefficient between observed and simulated data is 0.55 (Table 1). However, the deviation is larger in some areas, especially in the high elevation areas where the bias can reach 400 mm. Over YRB, the spatial characteristic of observed precipitation is more in east and less in west, more in south and less in north. The large deviation between observation and simulated precipitation mainly occurs near the Sichuan basin and Yun-Gui plateau with greatly undulate terrain. The spatial correlation coefficient between observed and simulated is 0.35 (Table 1).

**Table 1:** Comparison of observed and simulated precipitation before and after bias-correcting during 1961~2005 over the Tarim and Yangtze River Basin.

	Tarim River basin				Yangtze River basin			
	Bias/mm	RD/%	RMSE/mm	COR	Bias/mm	RD/%	RMSE/mm	COR
Observation and simulation	254.42	259.17	255.21	0.55	219.16	19.61	230.20	0.35
Observation and corrected simulation	−0.02	−0.02	22.89	1.00	−0.11	−0.01	113.00	1.00

Note: RD for the relative deviation, RMSE for the root mean square error, COR for the spatial correlation coefficient.

Based on the above analysis, the CMIP5 ensemble model can capture well in time series trend and spatial distribution over the TRB, while it is obviously better in data magnitude and seasonal rainfall distribution over the YRB. The Simulation of precipitation is significantly affected by elevation and topographic relief, and other climatic variables at the upper level should also be taken into account. The stations are few in the TRB, leading to the lack of original measured data used for CMIP5 simulation, which finally results in a large deviation of numerical simulation. Compared to the YRB in southeastern China, the observational data sequence is long and abundant, which is obviously affected by subtropical monsoon climate. At the same time, due to East West high lying low, the YRB is roughly three ladder-like distribution, resulting in a large deviation on simulating precipitation trends and spatial distribution.

#### Bias correction

For more reliable projections required for local climate impact assessment, we chose Equidistant Cumulative Distribution Functions^38^ (EDCDF) to correct the bias of the raw model output. Table 1 shows the comparison of observed and simulated precipitation before and after bias correction during 1961~2005 over the TRB and YRB. The precipitation bias between observed and simulated before correction (after correction) are 254.42 mm (−0.02 mm) over the TRB and 219.16 mm (−0.01 mm) over the YRB. In these two basins, compared with the uncorrected annual precipitation of the ensemble model, time series of corrected outputs have a smaller bias, RD and RMSE with observed data. And the spatial correlation coefficient between observation and corrected outputs even reach 1.00 in Fig. 3. After the bias correction, the monthly variation is also similar to the observation (Fig. 2). The precipitation concentration period of corrected simulation is same as the observation over two basins. The variation features and quantitative value are well captured by the corrected simulation data.

The EDCDF method can well correct the simulation of precipitation in both temporal and spatial scales over the TRB and YRB. In order to improve the reliability of the future precipitation, EDCDF method was also used in the future projection.

### Future projection

#### Annual precipitation

**Figure 4:**
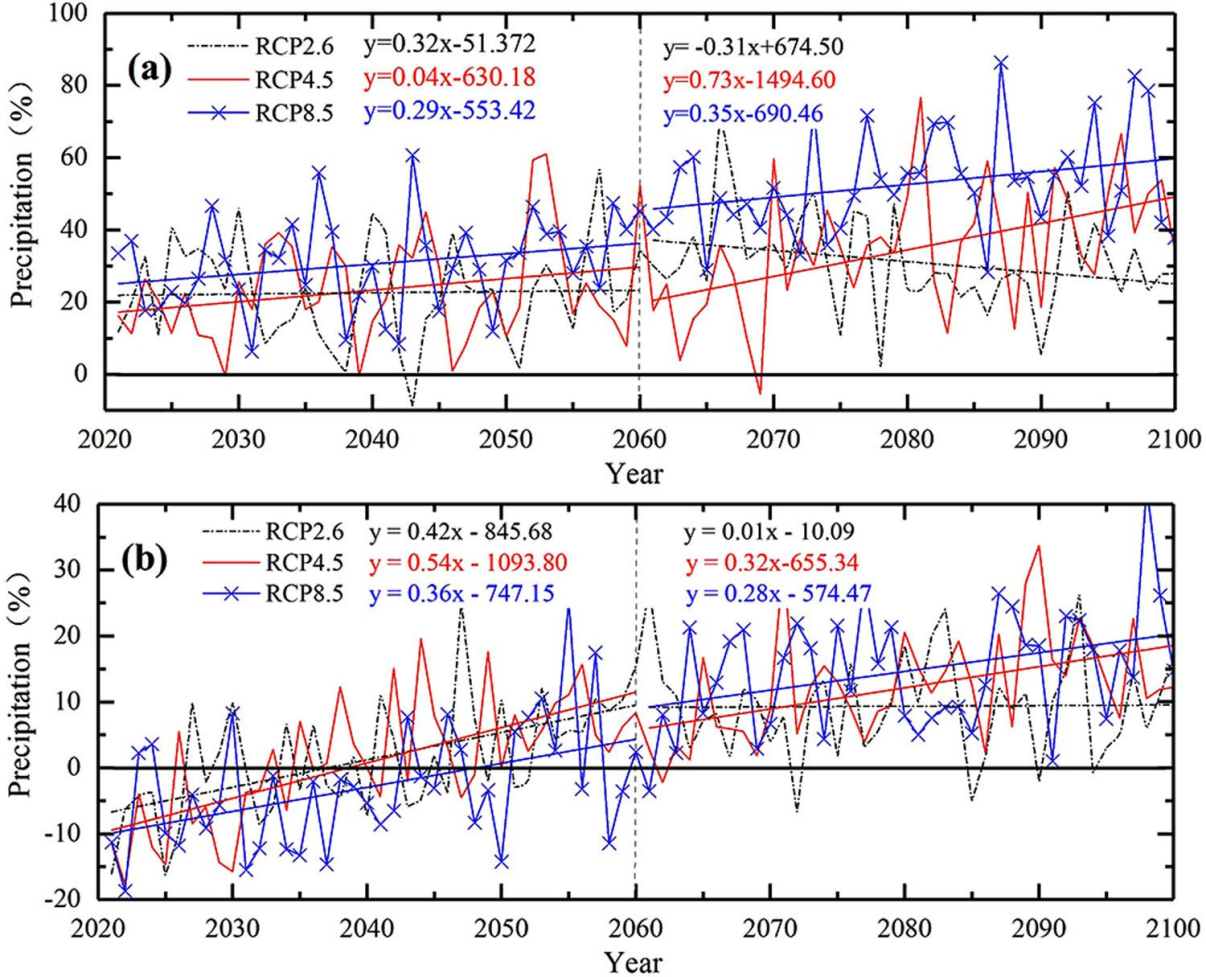
The temporal variation changes of precipitation during 2021~2100 under Scenario RCP2.6, Scenario RCP4.5, Scenario RCP8.5 (relative to those during 1961~2005) in the Tarim, (**a**) and Yangtze. (**b**) River Basin. (Note: Black dotted lines are the linear fitting for the annual precipitation under RCP2.6 during the 2021~2060 and 2061~2100, red lines are the linear fitting for the annual precipitation under RCP4.5 during the 2021~2060 and 2061~2100, blue lines are the linear fitting for the annual precipitation under RCP8.5 during the 2021~2060 and 2061~2100).

Based on the CMIP5 ensemble model after the bias correction, the annual precipitation was analyzed in 2021~2100 relative to 1961~2005 under RCP2.6, RCP4.5 and RCP8.5 (Fig. 4). Over the TRB, under RCP2.6, RCP4.5 and RCP8.5, the annual precipitation will increase by 26.83% (−8.67~72.4%), 29.14% (−5.49~76.70%) and 41.72% (6.28~86.38%) relatively, which shows obvious increasing trend. Precipitation trends in different periods (2021~2060 and 2061~2100) were further analyzed, the precipitation shows upward trend firstly (3.2 mm/10a) and then downward trend (−3.1 mm/10a) under RCP2.6, which will increase more obviously in 2061~2100 (7.3 mm/10a) than in 2021~2060 (0.4 mm/10a) under RCP4.5, and the precipitation fluctuation continues to increase steadily under RCP8.5. Over the YRB, under RCP2.6, RCP4.5 and RCP8.5, the annual precipitation will increase by 5.39% (−16.28~27.03%), 6.65% (−17.64~33.71%) and 5.99% (−18.72~42.61%) during 2021~2100 relative to 1961~2005. The annual precipitation in the early twenty-first century will reduce, while gradually increase in the middle and late twenty-first century. And the annual precipitation growth rate in 2061~2100 will decrease to some extent relative to 2021~2060. Under three emission scenarios, the annual precipitation trend lines keep similar, which indicates that the sensitivity of the annual precipitation to the emission scenario is not high over YRB, and the influence of climate change on precipitation will be further weakened. In general, the unified trend is that the fluctuation of precipitation will increase with the increase of scenarios emissions. However, the sensitivity of different hydrological factors to scenario emissions remains to be further verified.

**Figure 5:**
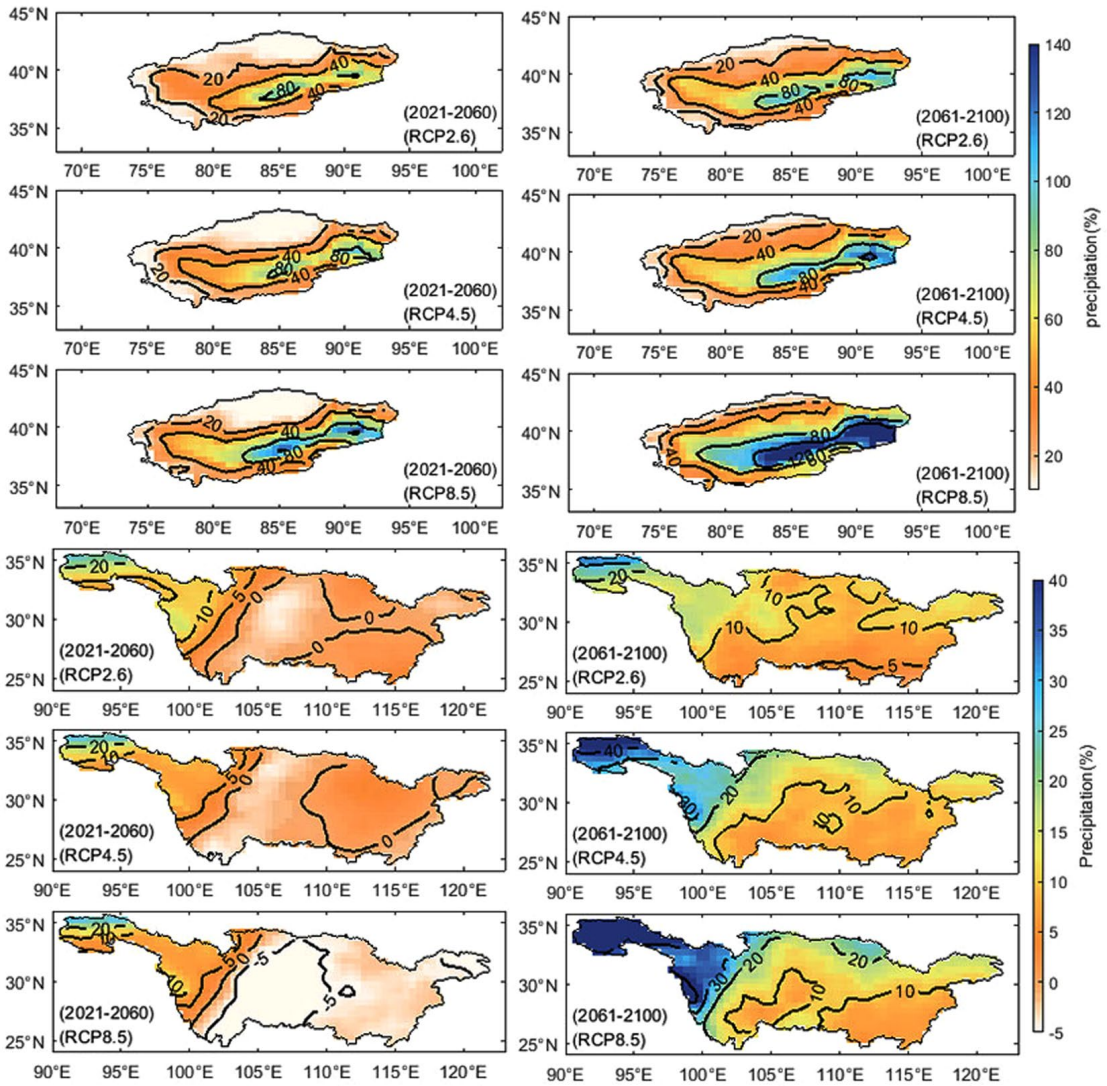
Spatial distribution percentage changes of precipitation (2021~2060 and 2061~2100) under Scenario RCP2.6, RCP4.5 and RCP8.5 (relative to those during 1961~2005) in the Tarim (top panel) and Yangtze River Basin (bottom panel). (Note: the maps were generated with data available from the Chinese Geospatial Data Cloud using Matlab (version R2016a; https://cn.mathworks.com/)).

In order to further analyze the spatial change of annual precipitation, Fig. 5 shows the spatial changes in the CMIP5 ensemble model after the bias correction over the TRB and YRB, as percentage change of simulated values in 2021~2060 and 2061~2100 relative to 1961~2005 under RCP2.6, RCP4.5 and RCP8.5. Over the TRB, precipitation shows no evident change near southern Tienshan Mountains, slightly upward trend near the Tarim River Mainstream Basin, sharp increase trend in the southern Tarim Basin and northern Altun Mountains and the increasing range of precipitation will increase with the increase of emission scenarios. In comparison to 2021~2060 (10~100%), more increase in annual precipitation is found under all scenarios in 2061~2100 (20~140%), especially in areas with greatly undulate terrain. Over the YRB, the annual precipitation in the source region of the YRB shows increasing trend (10~40%), the magnitude of the increase from east to west is increasing sequentially with the increase of the emission scenarios. For the decreasing trend in the early twenty-first century, although the annual precipitation is increasing under three emission scenarios, a decrease is also found over upper and middle reaches of the YRB during 2021~2060, especially for the RCP8.5 scenario. While during 2061~2100, under three emission scenarios, the precipitations keep similar change, with increase about 10% except in the source region of the YRB.

Compared with the YRB, the relative fluctuation range of annual precipitation has larger uncertainty over the TRB, which shows that the emission scenarios will have greater impact on precipitation with future climate change. This also indicates that the extreme events of precipitation will causes greater variation over the TRB.

#### Seasonal precipitation

The annual precipitation analysis can be used to obtain the inter-annual variation trend of precipitation, and the seasonal precipitation is more closely related to the production and life in reality^39^. Under RCP2.6, RCP4.5, RCP8.5, percentage change of the seasonal precipitation was summarized in 2021~2060 and 2061~2100 relative to those during 1961~2005 (Table 2). Although the future seasonal precipitation varies in two basins relative to that in the baseline period, the unified trend is that the change range of the seasonal precipitation in spring and winter is far greater than that in summer and autumn, which indicates that spring and winter precipitation is easier to be affected by climate change. Same as the annual precipitation, the change range of the seasonal precipitation over the TRB (5~150%) is greater than that over the YRB (0~15%).

**Table 2:** Changes of Seasonal Precipitation during 2021~2100 under Scenario RCP2.6, Scenario RCP4.5, Scenario RCP8.5 (relative to those during 1961~2005) in the Tarim and Yangtze River Basin.

Basins	Period	RCP	Spring precipitation/%	Summer precipitation/%	Autumn precipitation/%	Winter precipitation/%	Annual precipitation/%
Tarim River Basin		RCP2.6	52.14	6.41	24.86	70.94	22.59
	2021~2060	RCP4.5	60.37	3.48	27.03	79.82	23.46
		RCP8.5	68.01	9.82	32.30	98.89	30.68
		RCP2.6	64.75	16.08	27.06	70.23	31.07
	2061~2100	RCP4.5	88.90	7.56	28.98	126.34	34.82
		RCP8.5	141.28	6.94	44.84	210.09	52.77
Yangtze River Basin		RCP2.6	4.26	1.88	**−1.28**	**−3.87**	1.43
	2021~2060	RCP4.5	3.59	1.62	**−2.96**	**−1.12**	1.00
		RCP8.5	0.54	**−5.05**	**−3.89**	2.03	**−2.78**
		RCP2.6	14.09	6.15	8.23	14.96	9.36
	2061~2100	RCP4.5	21.27	7.44	8.51	20.05	12.30
		RCP8.5	29.66	11.53	5.33	7.40	14.76

Note: Bold text represents negative values of the percentage changes of precipitation during the 2021~2060 and 2061~2100 relative to 1961~2005.

The seasonal precipitation over the TRB suggests increasing trend as a whole in the next two periods, except for summer precipitation, all of which increases with the increase of the emission scenarios; Over the YRB, in addition to the spring precipitation, other three season precipitations decrease in different degrees during 2021~2060. While during 2061~2100, precipitation in spring and winter (7~30%) was slightly higher than that of summer and autumn (5~11%), there is no clear correlation between the seasonal precipitation and the emission scenarios. What mentioned above further verifies that precipitation over the YRB is not sensitive to future climate change.

**Figure 6:**
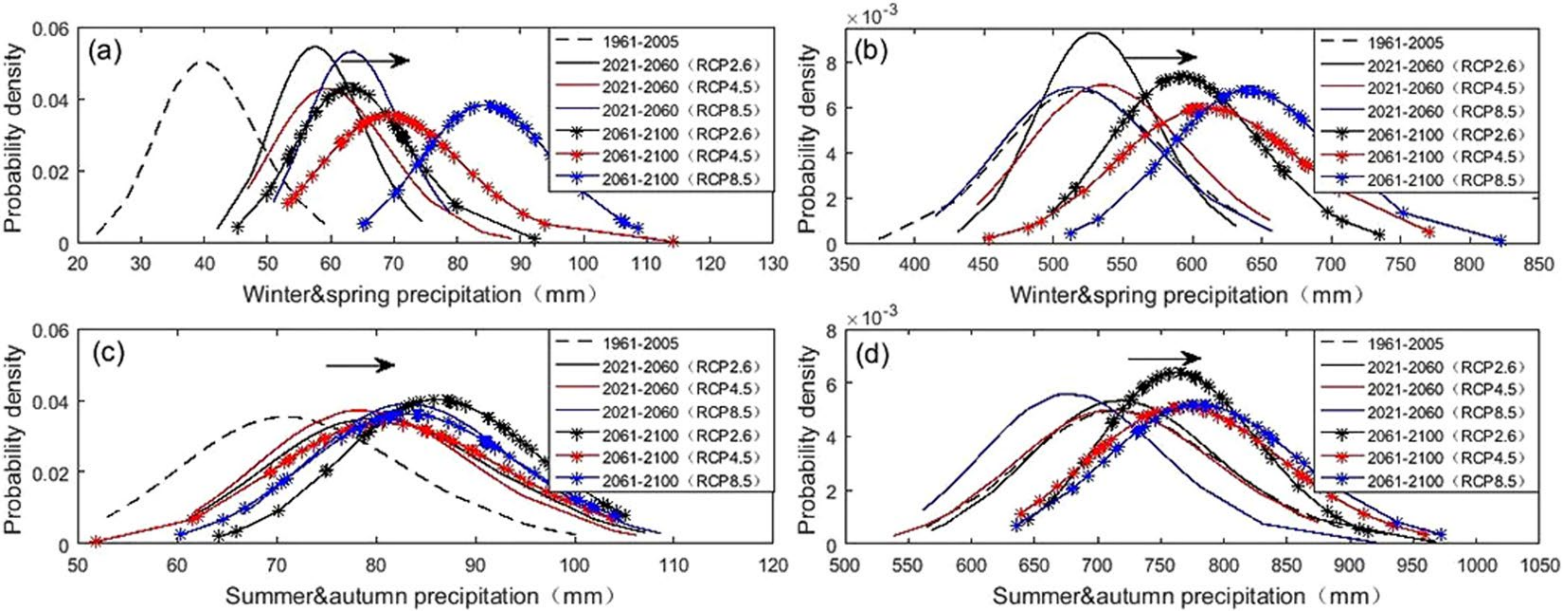
Comparison of probability distribution of winter/spring and summer/autumn precipitation from 2021 to 2060 and from 2061 to 2100 under RCP2.6,4.5 and 8.5 scenarios relative to 1961~2005 in the Tarim, (**a**,**c**) and Yangtze. (**b**,**d**) River Basin. (Note: the arrows represent the moving directions of future precipitation).

The percentage change of seasonal precipitation represents the influence degree of seasonal precipitation due to climate change, in order to have a more clear cognition on the temporal distribution of seasonal precipitation, the seasonal precipitation was further analyzed by empirical probability density^40^. As shown in Fig. 6, the shape of the probability density curve of models is shifting to right, which is similar to that in the baseline period in both two basins, of which the “right shift” range in spring and winter is significantly greater than that in summer and autumn. Over the TRB, “attenuation property” is detected in winter and spring precipitation distribution during 2061~2100 relative to that in the baseline period or 2021~2060, while summer and autumn precipitation is similar to that in baseline period, which indicates that winter and spring precipitation could be more variable in late twenty-first century. Over the YRB, the precipitation distribution in winter and spring during 2021~2060 and in summer and autumn during 2061~2100 are more concentrated under the RCP2.6, indicating that the uncertainty of precipitation distribution in the YRB will increases under the RCP2.6.

### Influence of geographical features

**Figure 7:**
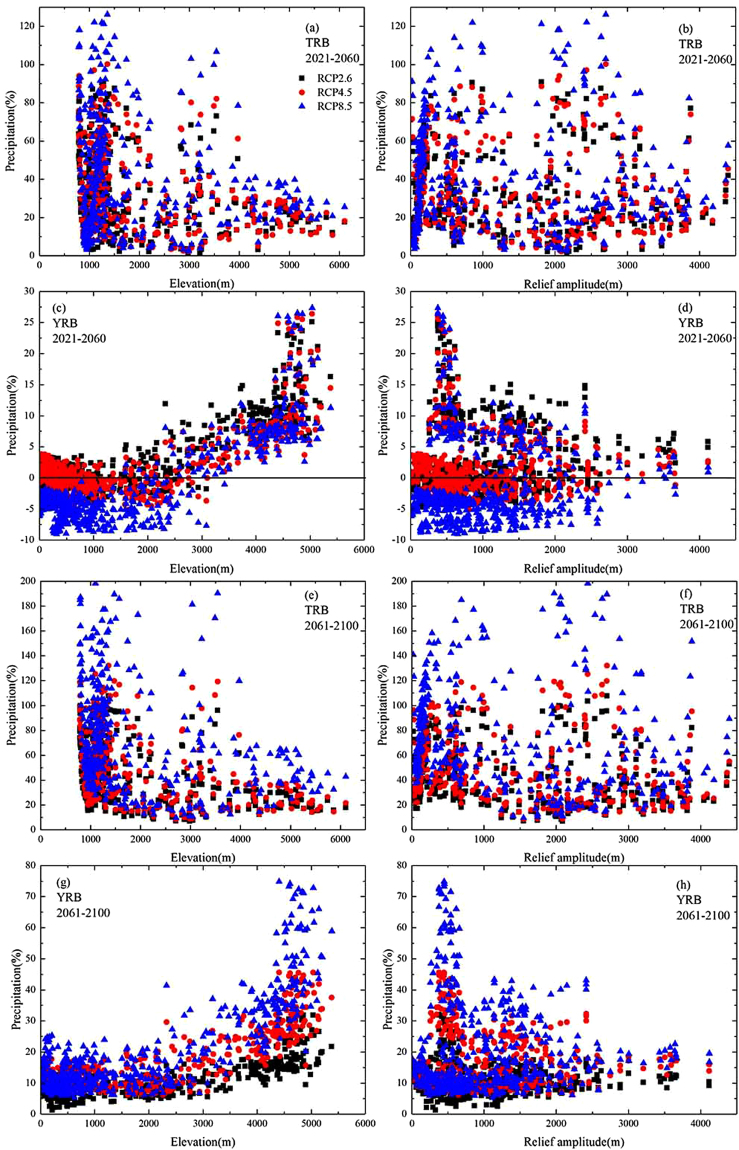
Changes of annual mean precipitation with altitude and relief amplitude during 2021–2060 (**a**–**d**) and 2021–2060 (**e**–**h**) under RCP2.6, 4.5 and 8.5 relative to 1961–2005 the Tarim and Yangtze River Basin (Note: TRB is for the Tarim River Basin, YRB is for the Yangtze River Basin).

Figure 7 displays the changes of annual precipitation with altitude and relief amplitude during 2021~2060 and 2061~2100 under RCP2.6, 4.5 and 8.5 relative to 1961~2005 over the TRB and YRB. Over the TRB, the maximum precipitation change mainly happens at lower elevations between 500~2000 m, located in most parts except mountains, among which the precipitation change under RCP8.5 is higher than the other two emission scenarios on the whole. And the change of precipitation decreases with altitude. In terms of greatly undulate terrain above 2000 m, the larger the relief amplitude of the study area is, the smaller the precipitation changes is. There is a larger range of precipitation change during 2061~2100 (0~200%) than during 2021~2060 (0~120%).

Over the YRB, contrary to the TRB, the change of precipitation increases with altitude, the maximum precipitation change mainly happens at higher elevations between 4000~5000 m, located in the source region of YRB on the Tibet Plateau. During 2021~2060, almost all grids have a certain increase in precipitation under the RCP2.6, except for some grids in the lower altitude area with the elevation between 2000~3000 m. While under the other two scenarios, the precipitation decreases with the elevation in the region below 3000 m. Compared with 2021~ 2060 (−10~30%), the precipitation tend to increase and all value changes are above 0 during 2061~2100 (0~80%). Same as what concluded in the TRB, the larger the relief amplitude of the study area is, the smaller the precipitation changes is, which could be applied to the whole basin.

Not only the elevation of study area, but also the relief amplitude has influence on simulating precipitation by CMIP5 data. However the topography of study area could have positive or negative effect on precipitation. There is no clear statistical relationship between precipitation simulation and elevation or relief amplitude in different basins. For the basin scale of 0.5° × 0.5° degrees, there is a negative correlation between the future precipitation change and the elevation over the TRB, while a positive correlation over the YRB. Based on the above rules, the future precipitation changes with elevation in the two basins can be verified.

## Discussion and Conclusions

Based on the precipitation of CN05.1 datasets, the ability of the CMIP5 ensemble model in simulating the historical climate and projecting the future climate change were evaluated in the TRB and YRB. Although with the bias from the GCMs limitations, particularly over the TRB, we could project the temporal and spatial distribution of precipitation based on the CMIP5 after bias correction. The statistical bias correction method (EDCDF) was shown to be useful for correcting the CMIP5 outputs. The uncertainty in the CMIP5 projections was explored in terms of elevation and relief amplitude. The major conclusions of this study were summarized as following.

The CMIP5 ensemble model behave differently on precipitation simulation in different basins. The model output can capture well in the trend of time series and the spatial distribution at the TRB. The annual precipitation increases according to the observed data (5.2 mm per decade) and the CMIP5 ensembles (3.7 mm per decade) at the TRB. The observed data shows that annual precipitation has an increasing trend (4.5 mm per decade) at the YRB, while the CMIP5 ensembles suggests a decreasing trend (−8.7 mm per decade) with a spatial correlation coefficient is 0.35. The model output seems providing better projection in magnitude and seasonal rainfall distribution at the YRB. The relative deviations of annual precipitation at the TRB and YRB are 259.17% and 19.61% respectively, likely due to the fact that few stations at the TRB provided less measurements used for the CMIP5 simulations. The simulated precipitation concentration period exists an offset over TRB, while seasonal variation of precipitation is well captured over the YRB, which may be resulted from the reason that precipitation over the YRB is obviously affected by subtropical monsoon climate.

Based on the CMIP5 ensemble model after the bias correction, under RCP2.6, RCP4.5 and RCP8.5, the increasing range of precipitation during 2021~2100 relative to 1961~2005 will increase with the increase of emission scenarios, especially over the TRB. The mean precipitation over TRB will increase by 26.83% (−8.67~72.4%), 29.14% (−5.49~76.70%) and 41.72% (6.28~86.38%) under three emission scenarios relatively, which shows obvious increasing trend. In comparison to 2021~2060 (10~100%), more increase in annual precipitation is found under all scenarios in 2061~2100 (20~140%). Precipitation shows sharp increasing trend in the southern Tarim Basin and northern Altun Mountains, which means that drought could be relived to some extent in these areas. The wide relative fluctuation range of annual precipitation indicates that the extreme events of precipitation will causes greater variation over the TRB. The annual precipitation over the YRB will increase by 5.39% (−16.28~27.03%), 6.65% (−17.64~33.71%) and 5.99% (−18.72~42.61%) under three emission scenarios relatively, which in the early twenty-first century will reduce, while gradually increase in the middle and late twenty-first century. For the decreasing trend in the early twenty-first century, although the mean precipitation is increasing, a decrease is also found over upper and middle reaches of the YRB during 2021~2060. While during 2061~2100, the precipitations keep similar change under three emission scenarios, with increase about 10% except in the source region of YRB (10~40%). The future precipitation change over YRB shows that water resources shortage will be eased until the late twenty-first century, which indicates that the “Drought-flood Abrupt Alternation” may occur in the middle twenty-first century.

The seasonal precipitation is influenced by varying degrees of impacts from different emission scenarios, which in spring and winter is far greater than that in summer and autumn based on the output of GCMs. Same as the annual precipitation, the change range of seasonal precipitation over the TRB (5~150%) is far greater than that over the YRB (0~15%). The increasing range of spring and winter precipitation (50~150%) is beneficial to the accumulation of glacier and the increase of water resources over the TRB, which is also positive to the development of agriculture. The extensive distribution of precipitation in spring and winter indicates that the precipitation in winter and spring is more changeable in the late twenty-first century. In addition to the spring precipitation, other three season precipitations decrease in different degrees during 2021~2060. While during 2061~2100, precipitation in spring and winter (7~30%) is slightly higher than that of summer and autumn (5~11%), the monitor effort need to be strengthened in case of spring flood disaster over the TRB.

The elevation and relief amplitude of the study area have influence on simulating precipitation with CMIP5 data. The maximum precipitation change mainly happens at lower elevations between 500~2000 m over the TRB, but at higher elevations between 4000~5000 m over the YRB. The change of precipitation will decrease with elevation over the TRB, while it increase over the YRB. In terms of the relief amplitude, the variation of precipitation will decrease with it over two basins at some extent. The elevation and relief amplitude of study area may increase the uncertainty of simulating precipitation by CMIP5 data.

## Materials and Methods

### Study area

**Figure 8:**
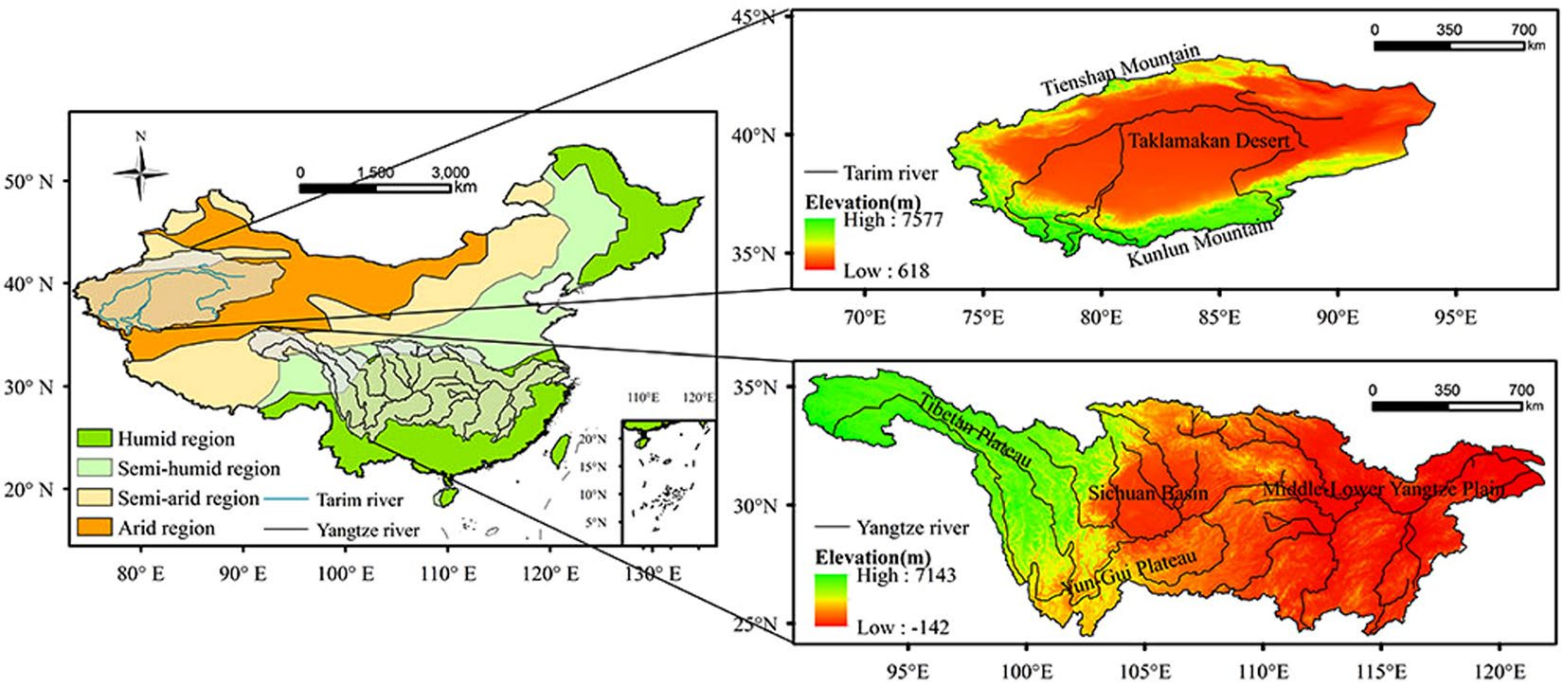
Sketch map of the Tarim and Yangtze River Basin (Note: the maps were generated with data available from the Chinese Geospatial Data Cloud using ESRI’s ArcGIS (version 10.1; http://www.gscloud.cn/)).

Located in northwestern China, the Tarim River Basin (TRB), lies between 73°E~94°E and 34°N~43°N and has a total drainage area of 1.02 million km^2^. The TRB is surrounded by the northern Tienshan Mountain and southern Kunlun Mountain on the edge of the Tibet Plateau^24,41^. In the center of TRB, the Taklamakan Desert, as the second largest shifting sand desert in world, accounts for over 33% of the total basin area^42,43^. With poor precipitation and strong evapotranspiration, TRB is a typical continental arid climate^44,45^. The Yangtze River Basin (YRB) is located in the southeastern China between 91°E~122°E and 25°N~35°N. The YRB has a total drainage area of 1.81 million km^2^, which originates from the Tibet Plateau at an elevation higher than 5000 m, flows eastwards into the East China Sea^46^. Except for the headwater region on the Qinghai-Tibet Plateau, YRB mainly belongs to the subtropical monsoon climate. Dry-Wet Climate zones are divided into the following four categories according to aridity index: arid area, semi-arid area, semi humid area and humid area (Fig. 8).

### Data

Twenty high-resolution global climate models in CMIP5 were selected to project future changes of precipitation in the TRB and YRB (Table 3). The historical climate simulations were extracted in the baseline period (1961~2005) to simulate the past precipitation, and the future projections were developed to evaluate the future climate changes for both near future (2021~2060) and long term future (2061~2100) under three RCP scenarios: RCP2.6, the low emission scenario, RCP4.5, the secondary stabilization scenario, and RCP8.5, the high emission scenarios^47^). Details on the models and datasets can be found or downloaded from the CMIP5 website (http://cmip-pcmdi.llnl.gov/cmip5/). Correspond to the grids number in the Table 3, different GCMs of the CMIP5 models have different resolutions. The observed data of daily precipitation used in this study was obtained from the high resolution gridded CN05.1 dataset (0.25° × 0.25°), which includes measurements at the 2400 stations in China^48^. In order to compare the observed and simulated precipitation, we interpolated the gridded data into 0.5° × 0.5° resolution with a bilinear interpolation. The outputs of GCMs were further integrated to the multi-model ensemble datasets using an arithmetic averaging approach. Digital Elevation models (DEM) of study areas were obtained from Shuttle Radar Topography Mission (SRTM) digital elevation data with 90 × 90 m grid cell resolution.

**Table 3:** Basic information on the 20 climate models in the Fifth Phase of the Coupled Model Inter-comparison Project (CMIP5).

I	Name	Country	Grids (Long-lat)	Id	Name	Country	Grids (Long-lat)
1	BCC-CSM1-1	China	128 × 64	11	IPSL-CM5A-LR	France	96 × 96
2	CNRM-CM5	France	256 × 128	12	IPSL-CM5A-MR	France	144 × 143
3	CSIRO-Mk3-6-0	Australia	192 × 96	13	MIROC5	Japan	256 × 128
4	FGOALS-g2	China	128 × 60	14	MIROC-ESM	Japan	128 × 64
5	GFDL-CM3	US	144 × 90	15	MIROC-ESM-CHEM	Japan	128 × 64
6	GFDL-ESM2G	US	144 × 90	16	MPI-ESM-LR	Germany	192 × 96
7	GFDL-ESM2M	US	144 × 90	17	MPI-ESM-MR	Germany	192 × 96
8	GISS-E2-H	US	144 × 90	18	MRI-CGCM3	Japan	320 × 160
9	GISS-E2-R	US	144 × 90	19	NCAR-CCSM4	US	288 × 192
10	HadGEM2-AO	Korea	192 × 145	20	NorESM1-M	Norway	144 × 96

### Methods

#### Simulation assessment

The precipitation simulation ability of CMIP5 was analyzed by comparing the CMIP5 data of the baseline period with the CN05.1 data. The following four indicators were used: the bias (BIAS), the relative deviation (RD), the root mean square error (RMSE) and the spatial correlation coefficient (COR).

1 $$BIAS=\frac{1}{t}\sum _{n=1}^{t}{({M}_{n}-{O}_{n})}^{2}=\bar{M}-\bar{O}$$

2 $$RD=|\frac{BIAS}{\bar{O}}|$$

3 $$RMSE={[\frac{1}{t}\sum _{n=1}^{t}{({M}_{n}-{O}_{n})}^{2}]}^{\frac{1}{2}}$$

4 $$COR=\frac{{\sum }_{i=1}^{N}({X}_{i\_fd1}-\,\overline{{X}_{i\_fd1}})({X}_{i\_fd2}-\,\overline{{X}_{i\_fd2}})}{\sqrt{{\sum }_{i=1}^{N}{({X}_{i\_fd1}-\overline{{X}_{i\_fd1}})}^{2}{\sum }_{i=1}^{N}{({X}_{i\_fd2}-\overline{{X}_{i\_fd2}})}^{2}}}\,$$

where *M*_*n*_ is the climate model datasets, *O*_*n*_ is the observed datasets, *t* is the number of sample, ${X}_{i\_fd1}({X}_{i\_fd2})$ is the value of field 1 (field 2) at grid point *i*, $\,\overline{{X}_{i\_fd1}}(\,\overline{{X}_{i\_fd2}})$ is the mean of all the grids points in field1 (field2), *N* is the total number of grid points in the field^49^.

#### Statistical bias correction

It is well known that climate model output data does not always accurately predict the climate variables. Statistical bias correction is generally used to obtain better statistical correspondence to observational data. The EDCDF^38^ was widely used as a bias correction method^12,50^, which incorporate and adjust the cumulative distribution functions (CDFs) of the model in the projection period on the basis of the difference between the model and observation CDFs in the baseline period. The EDCDF is defined as:

5 $${X}_{m-p,adj}={X}_{m-p}+{F}_{o-c}^{-1}[{F}_{m-p}({X}_{m-p})]-{F}_{m-c}^{-1}[{F}_{m-p}({X}_{m-p})]$$

where $X$ is the variables, $F$ is the Cumulative Distribution Function, ${\rm{o}}-{\rm{c}}$ is the historical observed value of the baseline period, while $m-c$ is the simulated value, $m-p$ is the predicated value of the future, and $\,{X}_{m-p,adj}$ is the bias corrected value.

In terms of monthly precipitation, a mixture of months with no rain and months with rain, especially for dry regions, can occur. A mixed gamma distribution could be set as the CDF of rainfall to explain the intermittent feature. The mixed CDF of monthly precipitation is defined as:

6 $$P(x)=(1-P)f(x)+PF(X)$$

where $P$ is the proportion of months with precipitation in total months, $f(x)$ is equal to 1 if there is precipitation, and it is equal to 0 if there is no precipitation, $F(x)$ is the CDF of the precipitation time series.

#### Uncertainty factors and impact

For CMIP5 projection, the uncertainty is a common feature in various regions^6^. In general, the uncertainty can be divided into three categories^13,16^: 1) Internal variability caused by random fluctuations of time series, which could potentially mask or enhance anthropogenic changes for about a decade. The internal climate fluctuations could be elevated by BIAS, RD, RMSE and COR. 2) The difference estimated by various models under the same emission scenario over the same area. A lots of studies^3,4,51^ confirmed that the uncertainties rising among different models is a sever issue for elevating future climate. In order to reduce the uncertainties and increase the reliability of prediction, the multi-model ensembles (MME) method is widely accepted for projection of climate change in the future^4^. 3) The uncertainty reflected in the variations of different emission scenarios for the future time. The emission scenarios provide information on possible development trajectories for the main forcing agents^52^, which could be analyzed by simulating and comparing the precipitation under three emission scenarios (RCP2.6, RCP4.5 and RCP8.5) in this paper.

Based on the uncertainty research, the influence of external changes over study areas on precipitation simulation should be considered. Based on the climatic conditions, relationship between precipitation change and the topographic features are further explored over the TRB and YRB respectively. In this paper, two topographic features, the elevation and relief amplitude, were used to analyze the internal relations. Relief amplitude refers to the difference of elevation between the highest and the lowest point in a particular area^53^. In order to get the grid DEM data, corresponding with precipitation data, the variables of elevation and relief amplitude on 0.5° × 0.5° resolution are obtained by using bilinear interpolation method with GIS resampling technique (Figure. S1 in the Supporting information).

## Electronic supplementary material

Supporting information
