# Supplementary material for: Study on the characteristics of future precipitation in response to external changes over arid and humid basins
Source: Sci Rep. 2017 Nov 9;7:15148. doi: 10.1038/s41598-017-15511-5 (PMC5680305; doi:10.1038/s41598-017-15511-5)
Supplement: Supplementary file 1 — Supporting information [file 41598_2017_15511_MOESM1_ESM.pdf]

Study on the characteristics of future precipitation in response to external changes over arid and humid basins

Lianqing Xue, Boli Zhu, Changbing Yang, Guanghui Wei, Xianyong Meng, Aihua Long, Guang Yang

Supplementary Information (SI)

SI Figure legends

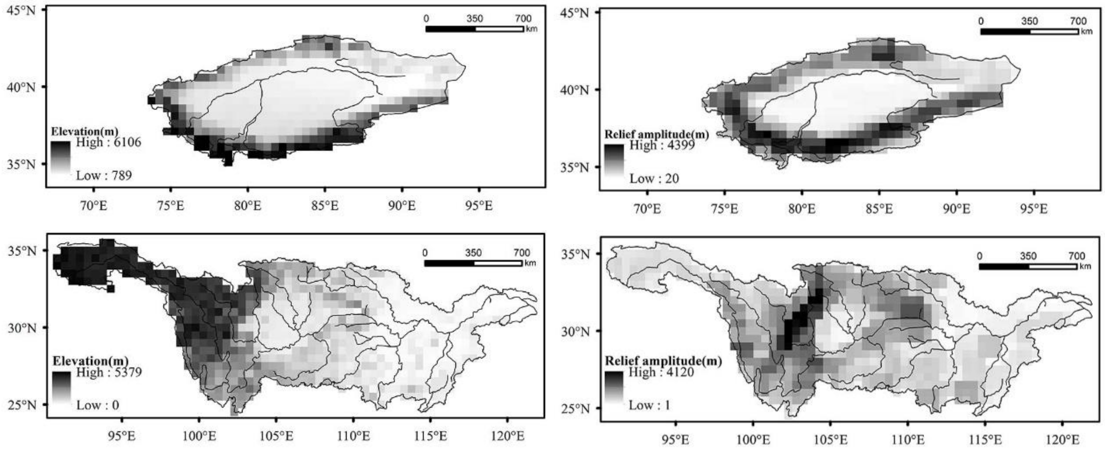

Figure.S1 the elevation and relief amplitude of the Tarim and Yangtze River Basin on 0.5 °×0.5 ° scale (Note: the maps were generated with data available from the Chinese Geospatial Data Cloud using ESRI's ArcGIS (version 10.1; http://www.gscloud.cn/).)
